# Diagnosis and management of parastomal pyoderma gangrenosum

**DOI:** 10.1093/gastro/got013

**Published:** 2013-04-18

**Authors:** Xian-rui Wu, Bo Shen

**Affiliations:** ^1^Department of Colorectal Surgery, the Cleveland Clinic Foundation,Cleveland, Ohio, USA and ^2^Department of Gastroenterology/Hepatology, the Cleveland Clinic Foundation, Cleveland, Ohio, USA

**Keywords:** inflammatory bowel disease, ileostomy, *pyoderma gangrenosum*, stoma

## Abstract

Parastomal *pyoderma gangrenosum* (PPG) is an unusual neutrophilic dermatosis characterized by painful, necrotic ulcerations occurring in the area surrounding an abdominal stoma. It typically affects young to middle-aged adults, with a slight female predominance. The underlying etiology for PPG remains enigmatic but aberrant immune response to injury may play a pivotal role. The reported risk factors for the development of PPG include the presence of extra-intestinal manifestations, autoimmune disorders and obesity, along with local factors, such as the presence of parastomal hernia or pressure ulcer. PPG can develop at any time after the stoma construction. The histopathological features of PPG are not pathognomonic and its diagnosis is mainly based on clinical features. The management of PPG often requires a multidisciplinary approach, with a combination of local wound care and systemic medications.

## INTRODUCTION

Initially described in 1930 by Brunsting [1], *pyoderma gangrenosum* (PG) is an unusual neutrophilic dermatosis, characterized by chronic, recurrent and painful cutaneous ulcerations. As an uncommon subtype, parastomal *pyoderma gangrenosum* (PPG), which occurs close to abdominal stomas, comprises about 15% of all cases of PG [2, 3]. Inflammatory bowel disease (IBD) was the first reported to be associated with PG and is the most frequently diagnosed underlying systemic disease for PPG [4–7] although other systemic disorders, such as diverticular disease, abdominal malignancy and neurological dysfunction, may also be associated with PPG [8]. PPG can occur in any kind of stoma, ranging from permanent or temporary jejunostomy, ileostomy or colostomy to loop- or end stomy, with the end ileostomy being the most common [9, 10]. It has been estimated that PPG occurs in 2.0–4.3% of patients who have had stoma surgery for IBD [4, 10–12], while its annual incidence rate in all abdominal stomas, for all indications, is reported to be 0.6% [8]. Multiple layers of evidence suggest that the incidence of PPG appears to be on the increase, probably due to careful investigation or increased awareness [13–15].

Though occurring at any age, PPG most commonly affects young to middle-aged adults, with a slight female predominance [9, 16, 17]. The health-related quality of life in patients with PPG can be seriously compromised, owing to consistent pain and poor application of stoma appliance. As in other types of PG, the etiology and pathogenesis of PPG is largely unknown, and risk factors are not well defined. Since the disease lacks characteristic histopathological features, PPG is a diagnosis of exclusion, primarily based on the clinical examination. Despite recent advances in medical therapy for IBD and its extra-intestinal manifestations (EIM), relapse of PPG following medical treatment is common and the long-term outcome for patients remains unpredictable [18–20].

## ETIOPATHOGENESIS AND RISK FACTORS

PG was initially thought to be caused by bacterial infection, disseminated to the skin from the bowel. However, no bacterial RNA was detected from the skin biopsies [21]. Although PG is estimated to be idiopathic in 25–50% of patients, an underlying aberrant immune response may be pivotal in the pathogenesis, as evidenced by its frequent association with autoimmune disorders [22, 23]. This might also help explain why pathergy (the development of new lesions or aggravation of existing ones following trivial trauma) occurs in 25–50% cases of PG [2, 24]. This hypothesis was supported by several later studies. Adachi Y *et al.* [25] found that humoral, cell-mediated and complement-dependent immune mechanisms were all abnormal in PG patients. The favorable response to therapy with cyclosporin or tacrolimus provides additional evidence for the role of abnormal T-cell function in the pathogenesis of PG [26–28]. The over-expression of interleukin (IL)-8 and IL-16, two potent chemotactic agents to leukocytes in PG tissues, were also reported [29, 30]. Neutrophil dysfunctions were shown to be associated with this disorder. PG is histologically characterized by the presence of inflammatory dermal infiltrates, composed of mature neutrophils [25, 31]. Although the neutrophils appear to be normal microscopically, a number of studies have demonstrated functional abnormality of these cells in PG [23, 25, 32]. Rare familial aggregation of PG has been reported, suggesting that genetic factors are etiologically important [33–36]. This notion is supported by the recent identification of mutations in the gene encoding the CD2-binding protein 1 in patients with ‘pyogenic sterile arthritis, PG and acne’ (PAPA), syndrome, a rare, autosomal dominant condition [37].

With the creation of a stoma, the skin around the area is assumed to be susceptible to the development of PPG. A study from France demonstrated that permanent stoma was significantly and independently associated with the development of PG after adjusting for other confounding factors [38]. The fact that the locations of the recurrent or newly emerging PPGs were often not at the same site as the old ones, also lent weight to this. Known topical factors accounting for the susceptibility of skin in this area include continual irritation from the leakage of bowel contents and inflammation caused by a stoma appliance [14, 39–42]. Inappropriate type and application of stoma appliance could also trigger PG by causing skin trauma through increased pressure on the skin [8]. The presence of a prominent parastomal hernia is another topical factor, because trauma could be the result, either from variations in skin tension or from increased friction against appliance and clothing [8]. A study by our group showed that patients with concurrent autoimmune disorders or a high body mass index (BMI) had a higher risk for the development of PPG [9]. Other reported risk factors for PPG include being female or African and having other EIMs of IBD [17, 38, 43].

## CLINICAL PRESENTATIONS

**Figure 1:**
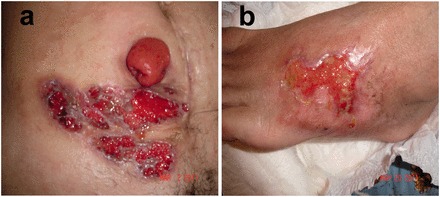
Pyoderma gangrenosum at the parastomal area and ankle with the same appearance.

There are four major types of PG, based on their clinical and histopathological features: ulcerative, pustular, bullous and vegetative [19]. PPGs are usually the ulcerative and/or vegetative ones, with ulcerative type the most frequent [13, 44]. The onset pattern of PPG is variable, it can develop at any time after stoma construction, ranging from weeks to several years [6, 8, 43, 45, 46]. The lesions can occur entirely or partly in the area of skin covered by the stoma appliance. Most ulcers are less than 3 cm in size; however, the ulcer can be multiple and an enormous ulcer of 30 cm has been reported [8, 39, 47]. The clinical course of PPG is not always associated with the severity of its underlying systemic diseases [48]. The lesion of PPG has a distinct clinical appearance, typically beginning as a deep-seated, painful nodule or as a superficial hemorrhagic pustule, either *de novo* or from minimal trauma. It then progresses into necrosis, with the lesion enlarged and broken down, undermining the surrounding skin and forming a burrowing, pyogenic ulcer crater. Established ulcers can be single or multiple lesions with irregular, erythematous margins, discharging purulent or hemorrhagic exudates (Fig. 1). Sometimes, bridges of normal-looking epithelium may traverse the ulcer base [2]. These ulcers are characterized by causing extreme pain (as opposed to itching). They can become destructive and rapidly expand by 1–2 cm in a single day [34]. The rapid progression is considered one of the hallmarks of the disease. When ulcers heal, they leave an unusual, web-like, cribriform, atrophic scar, which is very vulnerable to further breakdown through minor irritation or trauma.

## HISTOPATHOLOGY

Since its first description in 1930 [1], the histopathology of PG has been well defined. The histopathological findings may vary, depending on the area biopsied as well as the duration of the lesion [2, 49]. Skin biopsies taken from early lesions or the peripheral erythemous zone of established ulcers tend to reveal edema and massive neutrophil infiltration confined to the dermis. A prominent neutrophil infiltration is considered a histological feature for PG, which can be seen in active, untreated, expanding lesions. There may be mild-to- moderate perivascular lymphocyte infiltration associated with endothelial edema at the edge of the ulcer, which are features of vasculitis. Occasionally, thrombosis of small and medium- sized vessels, necrosis and extravasation of red blood cells are also seen. Biopsies taken later in the courses of ulceration often show neutrophil infiltration with ulceration, infarction and abscess formation. However, none of these histological features is pathognomonic and the primary objective of biopsy is to rule out other causes of ulceration, rather than to diagnose PG histologically [13, 18, 50–52].

## DIAGNOSIS AND DIFFERENTIAL DIAGNOSIS

There is no absolute diagnostic test for PG and the diagnosis is one that is based on a combined assessment of clinical and histological features. Several studies have proposed the diagnostic criteria for classic PG, including one from the Mayo Clinic [32, 53]. According to that study, the major diagnostic criteria were (i) rapid progression of a painful, necrolytic cutaneous ulcer with an irregular, violaceous and undermined border and (ii) other causes of cutaneous ulceration having been excluded. The minor diagnostic criteria included (i) history suggestive of pathergy or clinical finding of cribriform scarring, (ii) systemic diseases associated with PG, (iii) histopathological findings (sterile dermal neutrophilia, ± mixed inflammation, ± lymphocytic vasculitis) and (iv) rapid response to systemic steroid therapy. None of the criteria, either major or minor, can be used as a sole criterion; each can be seen in many disease states: however, the diagnosis of PG is indicated when both major criteria and at least two minor criteria are fulfilled.

The diagnostic evaluation of a patient presumed to have PPG has three objectives [17]: (i) to rule out other causes of parastomal ulcer, such as stitch abscess, contact dermatitis and wound infection, because micro-perforation from intestinal stoma may also lead to subcutaneous suppuration with resultant parastomal ulceration; (ii) to determine whether there is active underlying intestinal disease in the stoma and (iii) to identify any associated systemic disorders such as vasculitis, *erythema nodosum*, or similar lesions elsewhere which may provide a clue for the diagnosis of PPG. Although PG has no specific histological features, biopsy from the ulcer is still necessary and the need to rule out an alternative disease should override any fear of the occurrence of pathergy by performing a biopsy [18, 50]. Approximately 10% of patients with skin ulcers resulting from other causes were misdiagnosed as PG and evidence for alternative diagnoses was shown in the biopsies for most of the patients [50].

The importance of a prompt diagnosis of PPG cannot be overemphasized. Physicians should be aware of other disorders that mimic PPG when patients present with a chronic ulcerating skin lesion in the parastomal area. The differential diagnosis depends on the type and evolutionary stage of PPG. Generally, differential diagnoses include infectious diseases, malignancy, vasculitis and insect bites [18, 54]. A thorough review of history of the ulcer is usually helpful to rule out other courses and a careful gastrointestinal examination, such as colonoscopy or ileoscopy via stoma, will help to identify whether patients have underlying diseases, i.e. IBD. Swabs for smear and culture should be taken from the exudates and, when possible, directly from the ulcer. Biopsy may help exclude malignancy and vasculitis, as well as infection from fungi, mycobacteria, or parasites. Since ischemic changes also present as painful necrotic skin ulcers, ischemia-related skin ulcers and PG can be misdiagnosed. A study from a tertiary referral center in Switzerland showed that 16 (52%) of 31 hypertensive ischemic leg ulcers were referred with a suspected diagnosis of PG [55]. However, there is a paucity of data in the literature pertaining to the differential diagnosis between PPG and parastomal ischemia-related skin ulcers, probably due to the fact that the abdominal wall is an uncommon location for ischemic changes [56].

## MANAGEMENT

The proper management of PPG depends on correct diagnosis. If the skin ulceration is caused by other disorders—such as infection or malignancy—rather than PG, treatment directed at PG may produce substantial complications and *vice versa* [50]. Choices for the management of PPG rely largely on the experience of treating PG. Although it is still empiric in most cases, general consensus has been reached: once a PPG is diagnosed, a multidisciplinary approach, with the involvement of gastroenterologists, dermatologists, colorectal surgeons and stoma therapists is advocated. The goals of the treatment are to decrease local inflammation, to reduce the risk of infection and to control the contributing underlying disease [18, 57]. Therefore the underlying disease and stoma should also be meticulously examined and managed, except for the ulcer itself.

### Topical management

In mild cases, topical measures such as dressings, topical agents or intralesional injections may be sufficient to control the disease process (Table 1). Daily wound care should be performed through close liaison with a wound-care specialist. Moisture-retentive dressings appear to be superior to desiccative gauzes, in that they provide better pain control, induce collagen production, facilitate autolytic debridement and promote angiogenesis [58]. Furthermore, these occlusive dressings are useful as a means to avoid contaminating PPG with effluent. Cleansing of the wound with sterile saline is helpful, while the application of antibacterial agents—such as hydrogen peroxide or benzoyl peroxide—may also be useful [51, 59, 60]. Topical steroids and tacrolimus 0.3% preparations may be beneficial but care must be taken to exclude the possibility of concomitant infection [16, 61]. Patients whose diseases are in remission can try topical human platelet-derived growth factor if the process of re-epithelialization is slow [62, 63]. Intralesional injection of triamcinolone hexacetonide or cyclosporine has also been shown to be effective [64–66]. In general, the periphery of the lesion is injected, but the ulcer base may be injected too. Other topical therapies that may be used in controlling the inflammation and promoting wound healing include benzoyl peroxide, disodium cromoglycate, chlormethine, nicotine and 5-aminosalicylic acid [59, 62, 67–72].

**Table 1:** Management of PPG.

**Topical management**
Daily wound care. Moisture-retentive dressings. Topical agents, such as steroids or tacrolimus 0.3% preparations. Empiril antibiotic treatment can be applied if infection is suspected. Intralesional injection of triamcinolone hexacetonide or cyclosporine. Avoid ulcer debridement.
**Systemic management**
***Disease control***
Prednisone, 1 mg/kg per day. Hydrocortisone, 100 mg four times daily, intravenous injection. Methylprednisolone, 1 g/day (pulse therapy), intravenous injection.
***Maintenance therapy***
Dapsone, 100–300 mg per day. Minocycline, 100 mg twice daily. Cyclosporin, 3–5 mg/kg per day. Others: tacrolimus, 6-mercaptopurine, cyclophosphamide and chlorambucil
***Others***
Biologics: infliximab, adalimumab and etanercept. Plasma exchange. Human immunoglobulin infusion. Interferon-a therapy.
**General management**
Stoma care. Pain relief. Correction of anemia and malnutrition.

Although PPG is a non-infectious disease in origin, the ulcer can harbor superimposed bacterial infection. If infection is suspected, swabs for bacterial and fungal smear and culture should be taken and empiric antibiotic treatment that covers coliforms should be given immediately [48]. Surgical intervention for PPG—such as debridement—should be avoided, as pathergy can coincide in 25–50% cases of PG. The risk of developing recurrent and more refractory PG may be high [5].

### Systemic management

In patients who do not respond to topical or local therapies, who have a severe, rapid course, or who have active underlying disease (i.e. IBD), systemic management should be considered. Although there is no single therapy that can be efficacious in all cases, oral prednisone has been shown in the literature to be the most consistently successful agent for the treatment of PG [48]. Therapy with oral prednisone (1 mg/kg per day) is usually effective in controlling PG (Table 1) [73, 74]. The treatment should be continued until the lesions show evidence of healing and prolonged low-dose maintenance therapy is usually necessary in recurrent cases. Intravenous corticosteroid therapy (hydrocortisone 100 mg four times daily or methylprednisolone 1 g/day [pulse therapy]) for up to 5 days has also been reported to be successful (Table 1) [52, 75, 76]. Patients exposed to long-term use of prednisone are at risk of related side-effects: protecting agents, such as calcium, vitamin D and bisphosphonates can be used concomitantly [77]. Oral minocycline 100 mg twice daily may be of some benefit, usually as an adjunct to oral corticosteroid [2].

There are alternative ‘step-up’ therapies, the main purposes of which are twofold: (i) to reduce dependence on corticosteroids and (ii) to treat refractory disease. Dapsone and minocycline are the most frequently prescribed agents to provide a steroid-sparing effect [34, 54–56]. Oral dapsone 100–300 mg per day or minocycline 100 mg twice daily appear to be efficacious (Table 1). The mechanisms of action of these agents in the treatment of PG are not fully understood but they are related to its anti-microbial activity or anti-inflammatory effect. When corticosteroids fail, the most widely used alternative is cyclosporin [28, 74]. Several case reports and small case series demonstrate that most patients show clinical improvement within three weeks with a dose of 3–5 mg/kg per day and cyclosprin has been shown to be considerably more efficacious in the treatment of PG than azathioprine and methotrexate [26, 78, 79]. Other reported effective agents are tacrolimus, 6-mercaptopurine, cyclophosphamide, colchicine, clofazimine and chlorambucil [80–85].

Infliximab, an antibody against tumor necrosis factor α, has been shown to be efficacious in the management of PG. A randomized double blind, placebo-controlled trial by Brooklyn *et al.* compared 13 PG patients treated with infliximab with a group of 17 controls [3]. At 2 weeks, 46% of the infliximab group had responded, compared with 6% of the control group. Concerns about side-effects of infliximab, such as sepsis, have also been raised [48]. However, the benefits of infliximab outweigh the risks of its use and the agent has become the drug of choice in steroid-refractory PG. Although there are reports of refractory cases [86–88], adalimumab and etanercept are also thought to be effective biologic agents for PG[89–92]. Uses of plasma exchange, human immunoglobulin infusion and interferon-a therapy are also reported in more refractory PG (Table 1) [93–95].

### General management

Because of the persistent and recurrent nature of PG, a long-term maintenance therapy may be required. As a general measure, pain relief, correction of anemia, nutrition and management of associated disease are important. Stoma care, including use of an appropriate stoma appliance and prevention of leaks, also deserves attention (Table 1) [5]. Relocation of stoma should be contra-indicated except for other indications, such as parastomal herniation or stoma dysfunction.

## OUTCOMES

Despite advances in therapy, the long-term outcomes for PG patients remain unpredictable. Delayed and/or inappropriate treatment often necessitates hospitalization [96]. PG is a potentially lethal disease and the risk of death for PG patients was shown to be three three times higher than that for the general population [97]. Reported risk factors for poor prognosis include male gender, old age at onset and bullous type of PG, specifically when associated with malignant hematological disorders [18, 19]. The overall prognosis of PG without underlying disease, particularly in those patients who readily respond to treatment, is better compared with the idiopathic form [52, 98]. However, some studies showed that no difference was seen between patients with idiopathic and disease-associated PG, in terms of the recurrence and clinical outcomes [53, 99]. In contrast to PG, there are limited data on the outcomes of PPG. The existing studies seem to indicate a good prognosis in patients with PPG [9, 13, 43, 46]. A study with 20 cases of PPG demonstrated that all PPG ulcers healed completely after a median follow-up of 8 months (range: 1–41) [13]. Of these patients, ulcer resolution was achieved with medical therapy alone in 14 cases (70%). Similar findings were also noticed by our recent study [9], which showed that PPG either healed or improved in all of the 15 patients after a median follow-up of 12.8 months (interquartile range: 7.9–20.1). In our study, local wound care, by intra-lesional injection of corticosteroids and systemic corticosteroids or immunosuppressive agents, was administered in the majority of the patients (*n* = 13), resulting in an overall response rate of 73.3%. Biological agents were applied in the remaining four patients who failed the therapy and two of these patients had completely healed at the last follow-up. Potential risk factors affecting the prognosis of PPG are not well studied, but early diagnosis and early treatment are important for promoting the healing of PPG [10]. Further studies with a larger number of patients and longer-term follow-up are warranted to establish a consistent understanding of disease outcomes as a function of both disease variants and treatments.

## SUMMARY AND CONCLUSIONS

IBD is the most frequently diagnosed underlying systemic disease for PPG. All kinds of stomas can be involved, with end ileostomy being the most common one. Autoimmunity plays an important role in the pathogenesis of PPG, although the mechanism of this disease entity remains unclear. The risk factors for the occurrence of PPG are not well defined, but both local and systemic conditions can trigger its development. The primary objective of histopathological examination is to rule out other disorders and the diagnosis of PPG is mainly based on clinical findings. A multidisciplinary approach, involving gastroenterologists, dermatologists, colorectal surgeons and stomal therapists, is advocated. Both topical and systemic therapies are often required.

## FUNDING

The study was supported by research grants (to B.S.) from The Broad Foundation and The Crohn’s and Colitis Foundation of America.

**Conflict of interest:** The authors declare no financial conflict of interest.

## References

[1] Brunsting LA, Goeckerman WH, O’Leary PA (1930). Pyoderma gangrenosum: clinical and experimental observations in five cases occurring in adults. Arch Dermatol Syphilol.

[2] Brooklyn T, Dunnill G, Probert C (2006). Diagnosis and treatment of pyoderma gangrenosum. BMJ.

[3] Brooklyn TN, Dunnill MG, Shetty A (2006). Infliximab for the treatment of pyoderma gangrenosum: a randomised, double-blind, placebo-controlled trial. Gut.

[4] McGarity WC, Robertson DB, McKeown PP (1984). Pyoderma gangrenosum at the parastomal site in patients with Crohn's disease. Arch Surg.

[5] Kiran RP, O'Brien-Ermlich B, Achkar JP (2005). Management of peristomal pyoderma gangrenosum. Dis Colon Rectum.

[6] Shabbir J, Britton DC (2010). Stoma complications: a literature overview. Colorectal Dis.

[7] Last M, Fazio V, Lavery I, Jagelman D (1984). Conservative management of paraileostomy ulcers in patients with Crohn's disease. Dis Colon Rectum.

[8] Lyon CC, Smith AJ, Beck MH (2000). Parastomal pyoderma gangrenosum: clinical features and management. J Am Acad Dermatol.

[9] Wu XR, Mukewar S, Kiran RP (2013). Risk factors for peristomal pyoderma gangrenosum complicating inflammatory bowel disease. J Crohns Colitis.

[10] Funayama Y, Kumagai E, Takahashi K (2009). Early diagnosis and early corticosteroid administration improves healing of peristomal pyoderma gangrenosum in inflammatory bowel disease. Dis Colon Rectum.

[11] Brady E (1999). Severe peristomal pyoderma gangrenosum: a case study. J Wound Ostomy Continence Nurs.

[12] Uchino M, Ikeuchi H, Matsuoka H (2012). Clinical features and management of parastomal pyoderma gangrenosum in inflammatory bowel disease. Digestion.

[13] Sheldon DG, Sawchuk LL, Kozarek RA, Thirlby RC (2000). Twenty cases of peristomal pyoderma gangrenosum: diagnostic implications and management. Arch Surg.

[14] Ahmadi S, Powell FC (2005). Pyoderma gangrenosum: uncommon presentations. Clin Dermatol.

[15] Hanley J (2011). Effective management of peristomal pyoderma gangrenosum. Br J Nurs.

[16] Bennett ML, Jackson JM, Jorizzo JL (2000). Pyoderma gangrenosum. A comparison of typical and atypical forms with an emphasis on time to remission. Case review of 86 patients from 2 institutions. Medicine (Baltimore).

[17] Tjandra JJ, Hughes LE (1994). Parastomal pyoderma gangrenosum in inflammatory bowel disease. Dis Colon Rectum.

[18] Ruocco E, Sangiuliano S, Gravina AG (2009). Pyoderma gangrenosum: an updated review. J Eur Acad Dermatol Venereol.

[19] Conrad C, Trueb RM (2005). Pyoderma gangrenosum. J Dtsch Dermatol Ges.

[20] Miller J, Yentzer BA, Clark A (2010). Pyoderma gangrenosum: a review and update on new therapies. J Am Acad Dermatol.

[21] Crowson AN, Nuovo GJ, Mihm MC, Magro C (2003). Cutaneous manifestations of Crohn's disease, its spectrum and its pathogenesis: intracellular consensus bacterial 16S rRNA is associated with the gastrointestinal but not the cutaneous manifestations of Crohn's disease. Hum Pathol.

[22] Guenova E, Teske A, Fehrenbacher B (2011). Interleukin 23 expression in pyoderma gangrenosum and targeted therapy with ustekinumab. Arch Dermatol.

[23] Ahronowitz I, Harp J, Shinkai K (2012). Etiology and management of pyoderma gangrenosum: a comprehensive review. Am J Clin Dermatol.

[24] Perry HO (1969). Pyoderma gangrenosum. South Med J.

[25] Adachi Y, Kindzelskii AL, Cookingham G (1998). Aberrant neutrophil trafficking and metabolic oscillations in severe pyoderma gangrenosum. J Invest Dermatol.

[26] Schofer H, Baur S (2002). Successful treatment of postoperative pyoderma gangrenosum with cyclosporin. J Eur Acad Dermatol Venereol.

[27] Weichert G, Sauder DN (1998). Efficacy of tacrolimus (FK 506) in idiopathic treatment-resistant pyoderma gangrenosum. J Am Acad Dermatol.

[28] Friedman S, Marion JF, Scherl E (2001). Intravenous cyclosporine in refractory pyoderma gangrenosum complicating inflammatory bowel disease. Inflamm Bowel Dis.

[29] Oka M, Berking C, Nesbit M (2000). Interleukin-8 overexpression is present in pyoderma gangrenosum ulcers and leads to ulcer formation in human skin xenografts. Lab Invest.

[30] Yeon HB, Lindor NM, Seidman JG, Seidman CE (2000). Pyogenic arthritis, pyoderma gangrenosum and acne syndrome maps to chromosome 15q. Am J Hum Genet.

[31] Vignon-Pennamen MD (2000). The extracutaneous involvement in the neutrophilic dermatoses. Clin Dermatol.

[32] Su WP, Davis MD, Weenig RH (2004). Pyoderma gangrenosum: clinicopathologic correlation and proposed diagnostic criteria. Int J Dermatol.

[33] Al-Rimawi HS, Abuekteish FM, Daoud AS, Oboosi MM (1996). Familial pyoderma gangrenosum presenting in infancy. Eur J Pediatr.

[34] Khandpur S, Mehta S, Reddy BS (2001). Pyoderma gangrenosum in two siblings: a familial predisposition. Pediatr Dermatol.

[35] Alberts JH, Sams HH, Miller JL, King LE (2002). Familial ulcerative pyoderma gangrenosum: a report of 2 kindred. Cutis.

[36] Goncalves J, Capon Degardin N, Laurent F (2002). Familial pyoderma gangrenosum following a mammoplasty reduction: a case report. Ann Chir Plast Esthet.

[37] Wise CA, Gillum JD, Seidman CE (2002). Mutations in CD2BP1 disrupt binding to PTP PEST and are responsible for PAPA syndrome, an autoinflammatory disorder. Hum Mol Genet.

[38] Farhi D, Cosnes J, Zizi N (2008). Significance of erythema nodosum and pyoderma gangrenosum in inflammatory bowel diseases: a cohort study of 2402 patients. Medicine (Baltimore).

[39] Prystowsky JH, Kahn SN, Lazarus GS (1989). Present status of pyoderma gangrenosum. Review of 21 cases. Arch Dermatol.

[40] Hill MP, Vigneaud H, Zukervar P, Perrot H (1991). Parastomal pyoderma gangrenosum. 3 new cases. Ann Dermatol Venereol.

[41] Marzano AV, Cugno M, Trevisan V (2010). Role of inflammatory cells, cytokines and matrix metalloproteinases in neutrophil-mediated skin diseases. Clin Exp Immunol.

[42] Vornehm ND, Kelley SR, Ellis BJ (2011). Parastomal small bowel evisceration as a result of parastomal pyoderma gangrenosum in a patient with Crohn's disease. Am Surg.

[43] Cairns BA, Herbst CA, Sartor BR (1994). Peristomal pyoderma gangrenosum and inflammatory bowel disease. Arch Surg.

[44] Dabade TS, Davis MD (2011). Diagnosis and treatment of the neutrophilic dermatoses (pyoderma gangrenosum; Sweet's syndrome). Dermatol Ther.

[45] Hellman J, Lago CP (1990). Dermatologic complications in colostomy and ileostomy patients. Int J Dermatol.

[46] Hughes AP, Jackson JM, Callen JP (2000). Clinical features and treatment of peristomal pyoderma gangrenosum. JAMA.

[47] Hoffman MD (2007). Inflammatory ulcers. Clin Dermatol.

[48] Campbell S, Cripps S, Jewell DP (2005). Therapy Insight: pyoderma gangrenosum: old disease, new management. Nat Clin Pract Gastroenterol Hepatol.

[49] Su WP, Schroeter AL, Perry HO, Powell FC (1986). Histopathologic and immunopathologic study of pyoderma gangrenosum. J Cutan Pathol.

[50] Weenig RH, Davis MD, Dahl PR, Su WP (2002). Skin ulcers misdiagnosed as pyoderma gangrenosum. N Engl J Med.

[51] Callen JP (1998). Pyoderma gangrenosum. Lancet.

[52] Powell FC, Su WP, Perry HO (1996). Pyoderma gangrenosum: classification and management. J Am Acad Dermatol.

[53] Von den Driesch P (1997). Pyoderma gangrenosum: a report of 44 cases with follow-up. Br J Dermatol.

[54] Ruhl AP, Ganz JE, Bickston SJ (2007). Neutrophilic folliculitis and the spectrum of pyoderma gangrenosum in inflammatory bowel disease. Dig Dis Sci.

[55] Hafner J, Nobbe S, Partsch H (2010). Martorell hypertensive ischemic leg ulcer: a model of ischemic subcutaneous arteriolosclerosis. Arch Dermatol.

[56] Dean SM (2008). Atypical ischemic lower extremity ulcerations: a differential diagnosis. Vasc Med.

[57] Fraccalvieri M, Fierro MT, Salomone M (2012). Gauze-based negative pressure wound therapy: a valid method to manage pyoderma gangrenosum?. Int Wound J.

[58] Fonder MA, Lazarus GS, Cowan DA (2008). Treating the chronic wound: a practical approach to the care of non-healing wounds and wound care dressings. J Am Acad Dermatol.

[59] Nguyen LQ, Weiner J (1977). Treatment of pyoderma gangrenosum with benzoyl peroxide. Cutis.

[60] Baumgart DC, Wiedenmann B, Dignass AU (2004). Successful therapy of refractory pyoderma gangrenosum and periorbital phlegmona with tacrolimus (FK506) in ulcerative colitis. Inflamm Bowel Dis.

[61] Lyon CC, Stapleton M, Smith AJ (2001). Topical tacrolimus in the management of peristomal pyoderma gangrenosum. J Dermatolog Treat.

[62] Braun-Falco M, Stock K, Ring J, Hein R (2002). Topical platelet-derived growth factor accelerates healing of myelodysplastic syndrome-associated pyoderma gangrenosum. Br J Dermatol.

[63] Kurtz MP, Svensson E, Heimann TM (2011). Use of platelet-derived growth factor for delayed perineal wound healing in patients with inflammatory bowel disease: a case series. Ostomy Wound Manage.

[64] Goldstein F, Krain R, Thornton JJ (1985). Intralesional steroid therapy of pyoderma gangrenosum. J Clin Gastroenterol.

[65] Jennings JL (1983). Pyoderma gangrenosum: successful treatment with intralesional steroids. J Am Acad Dermatol.

[66] V'Lckova-Laskoska MT, Laskoski DS, Caca-Biljanovska NG, Darkoska JS (1999). Pyoderma gangrenosum successfully treated with cyclosporin A. Adv Exp Med Biol.

[67] Tamir A, Landau M, Brenner S (1996). Topical treatment with 1% sodium cromoglycate in pyoderma gangrenosum. Dermatology.

[68] Patel GK, Rhodes JR, Evans B, Holt PJ (2004). Successful treatment of pyoderma gangrenosum with topical 0.5% nicotine cream. J Dermatolog Treat.

[69] Sanders CJ, Hulsmans RF (1993). Successful treatment of pyoderma gangrenosum with topical 5-aminosalicylic acid. Cutis.

[70] Powell FC, O'Kane M (2002). Management of pyoderma gangrenosum. Dermatol Clin.

[71] Tsele E, Yu RC, Chu AC (1992). Pyoderma gangrenosum: response to topical nitrogen mustard. Clin Exp Dermatol.

[72] Wenzel J, Gerdsen R, Phillipp-Dormston W (2002). Topical treatment of pyoderma gangraenosum. Dermatology.

[73] Powell FC, Collins S (2000). Pyoderma gangrenosum. Clin Dermatol.

[74] Reichrath J, Bens G, Bonowitz A, Tilgen W (2005). Treatment recommendations for pyoderma gangrenosum: an evidence-based review of the literature based on more than 350 patients. J Am Acad Dermatol.

[75] Futami H, Kodaira M, Furuta T (1998). Pyoderma gangrenosum complicating ulcerative colitis: Successful treatment with methylprednisolone pulse therapy and cyclosporine. J Gastroenterol.

[76] Galun E, Flugelman MY, Rachmilewitz D (1986). Pyoderma gangrenosum complicating ulcerative colitis: successful treatment with methylprednisolone pulse therapy and dapsone. Am J Gastroenterol.

[77] Kumar R (2001). Glucocorticoid-induced osteoporosis. Curr Opin Nephrol Hypertens.

[78] Matis WL, Ellis CN, Griffiths CE, Lazarus GS (1992). Treatment of pyoderma gangrenosum with cyclosporine. Arch Dermatol.

[79] Carp JM, Onuma E, Das K, Gottlieb AB (1997). Intravenous cyclosporine therapy in the treatment of pyoderma gangrenosum secondary to Crohn's disease. Cutis.

[80] Wollina U (2007). Pyoderma gangrenosum: a review. Orphanet J Rare Dis.

[81] Pari T, George S, Jacob M (1996). Malignant pyoderma responding to clofazimine. Int J Dermatol.

[82] Kontochristopoulos GJ, Stavropoulos PG, Gregoriou S, Zakopoulou N (2004). Treatment of pyoderma gangrenosum with low-dose colchicine. Dermatology.

[83] Callen JP, Case JD, Sager D (1989). Chlorambucil: an effective corticosteroid-sparing therapy for pyoderma gangrenosum. J Am Acad Dermatol.

[84] Zonana-Nacach A, Jimenez-Balderas FJ, Martinez-Osuna P, Mintz G (1994). Intravenous cyclophosphamide pulses in the treatment of pyoderma gangrenosum associated with rheumatoid arthritis: report of 2 cases and review of the literature. J Rheumatol.

[85] Maldonado N, Torres VM, Mendez-Cashion D (1968). Pyoderma gangrenosum treated with 6-mercaptopurine and followed by acute leukemia. J Pediatr.

[86] Kleinpenning MM, Langewouters AM, Van de Kerkhof PC, Greebe RJ (2011). Severe pyoderma gangrenosum unresponsive to etanercept and adalimumab. J Dermatolog Treat.

[87] Poulin Y (2009). Successful treatment of hidradenitis suppurativa with infliximab in a patient who failed to respond to etanercept. J Cutan Med Surg.

[88] Hinterberger L, Muller CS, Vogt T, Pfohler C (2012). Adalimumab: a treatment option for pyoderma gangrenosum after failure of systemic standard therapies. Dermatol Ther (Heidelb).

[89] Lipka S, Katz S, Ginzburg L (2012). Massive pyoderma gangrenosum in a 77-year-old female with Crohn's disease responsive to adalimumab. J Crohns Colitis.

[90] Carinanos I, Acosta MB, Domenech E (2011). Adalimumab for pyoderma gangrenosum associated with inflammatory bowel disease. Inflamm Bowel Dis.

[91] Kim FS, Pandya AG (2012). The use of etanercept in the treatment of peristomal pyoderma gangrenosum. Clin Exp Dermatol.

[92] Rogge FJ, Pacifico M, Kang N (2008). Treatment of pyoderma gangrenosum with the anti-TNF alpha drug - etanercept. J Plast Reconstr Aesthet Surg.

[93] Gerard A, Schooneman F, Voiriot P (1988). Pyoderma gangrenosum: treatment with plasma exchange (4 cases). Ann Med Interne (Paris).

[94] Jolles S, Hughes J (2006). Use of IVIG in the treatment of atopic dermatitis, urticaria, scleromyxedema, pyoderma gangrenosum, psoriasis and pretibial myxedema. Int Immunopharmacol.

[95] Smith JB, Shenefelt PD, Soto O, Valeriano J (1996). Pyoderma gangrenosum in a patient with cryoglobulinemia and hepatitis C successfully treated with interferon alfa. J Am Acad Dermatol.

[96] Saracino A, Kelly R, Liew D, Chong A (2011). Pyoderma gangrenosum requiring inpatient management: a report of 26 cases with follow up. Australas J Dermatol.

[97] Langan SM, Groves RW, Card TR, Gulliford MC (2012). Incidence, mortality and disease associations of pyoderma gangrenosum in the United Kingdom: a retrospective cohort study. J Invest Dermatol.

[98] Callen JP, Jackson JM (2007). Pyoderma gangrenosum: an update. Rheum Dis Clin North Am.

[99] Mlika RB, Riahi I, Fenniche S (2002). Pyoderma gangrenosum: a report of 21 cases. Int J Dermatol.

